# When Less Is More: Investigating Factors Influencing the Distraction Effect of Virtual Reality From Pain

**DOI:** 10.3389/fpain.2021.800258

**Published:** 2022-01-05

**Authors:** Katharina Barcatta, Elisabeth Holl, Layla Battistutta, Marian van der Meulen, Katharina M. Rischer

**Affiliations:** Department of Behavioral and Cognitive Sciences, University of Luxembourg, Esch-sur-Alzette, Luxembourg

**Keywords:** virtual reality, distraction from pain, cognitive load, emotional distress, executive functions

## Abstract

Virtual reality (VR) is a powerful method of redirecting attention away from pain. Yet, little is known about which factors modulate the size of this distraction effect. The aim of this study was to investigate the role of cognitive load and inter-individual differences in the cognitive and affective domain on heat pain thresholds during a VR game. Ninety healthy participants (mean age ± SD: 23.46 ± 3.28; 50% identified as male and 50% as female) played a low and high load version of a VR game while heat pain thresholds and heart rate were recorded. The effects of cognitive load were assessed by computing the difference in pain thresholds between the high and low load condition for each participant. In addition, we computed the difference in heart rate variability (HRV) measures between both conditions to explore whether these would be correlated with the difference in heat pain thresholds. Prior to the VR session, participants completed questionnaires about their emotional distress, pain-related cognitions, and different executive functioning tasks. Contrary to our expectations, not all participants benefitted from a higher load in terms of distraction from pain. Logistic regression analysis revealed that participants who reported more emotional distress were more likely to exhibit higher pain thresholds in the low relative to the high load condition. Accordingly, these participants tended to show marginally higher HRV in the low compared to the high load condition. Our study demonstrates that the potential benefits of an increased cognitive load in VR on pain sensitivity depends on individual differences in affective state.

## Introduction

Virtual reality (VR) has proven to be a powerful tool for attentional diversion and is increasingly used in clinical settings for pain management (1–4). The distractive, hypoalgesic effects of VR are assumed to result from a competition for limited shared attentional resources between the immersive, sensory properties of VR and incoming nociceptive signals (5).

Previous research suggests that several factors influence the efficacy of cognitive distraction from pain in non-VR settings, yet little is known about their role in VR (3). For example, tasks placing a high demand on central executive resources are particularly effective in diverting attention away from pain (6, 7). However, studies investigating the effects of increased task difficulty in VR often rely on the manipulation of game settings, such as the speed or number of hit targets, and are therefore likely to be confounded by the participant's gaming experience and motor coordination skills (8–10).

Other factors that may influence pain modulation are stress level (11–13) and negative pain-related cognitions, such as the tendency to catastrophize about pain (14, 15). Furthermore, a growing body of studies suggests that better executive functions (EFs), specifically cognitive inhibition abilities, are associated with reduced pain sensitivity (16, 17) and may play a role in cognitive distraction from pain (18). However, while there is a growing body of research on the effects of virtual reality on pain, and (pain-related) anxiety and distress (19, 20), it is less well known how these factors influence the efficacy of virtual reality in reducing pain.

The aim of the present study was to examine whether increasing cognitive load through the implementation of a memory task in VR would enhance the hypoalgesic effect of VR when compared to a low load condition, as demonstrated in non-VR settings. Furthermore, we investigated whether emotional distress, pain-related cognitions and executive functions would predict the likelihood to benefit from the higher cognitive load. To account for individual differences in previous gaming experience, we also assessed gaming skills and simulator sickness symptoms.

We expected that an additional load in the VR game would lead to decreased pain sensitivity, i.e., higher heat pain thresholds, when compared to a low load condition. We also expected that individuals with better executive functions would be more likely to benefit from the additional cognitive load, as they might be able to better ignore the pain and selectively focus attention on the VR and memory task. We had no clear hypotheses about the influence of pain-related cognitions or emotional distress on the effect of an additional cognitive load. Throughout each condition, we recorded electrocardiograms to obtain heart rate variability (HRV) measures to explore whether pain modulation by cognitive load would be associated with changes in HRV. We had no clear hypothesis about the direction of the association: while a recent study by Colloca et al. (21) reported that decreased pain sensitivity in a relaxing VR environment correlated with higher HRV, pain relief by cognitive distraction has been associated with decreased HRV (22), which is consistent with studies that have linked higher mental stress to lower HRV (23, 24). In the present study, we wanted to assess whether changes in HRV would also reflect changes in pain thresholds in a more cognitively challenging VR environment.

## Materials and Methods

### Participants

A total of 101 participants were recruited via student forums, posters, social media, and a radio interview to reach participants outside of the university environment and tested in the MExLab at the University of Luxembourg between September and December 2019. Participants were healthy young adults between 18 and 35 years old and fluent in either German or English. Exclusion criteria were acute or chronic pain, intake of pain medication, a diagnosis of photosensitive epilepsy and neurodermatitis or other skin-related medical conditions on the participants' non-dominant leg (details about dropouts can be found in Descriptive Statistics). Each participant was compensated with a 25€ gift voucher and optional course credit. The study was approved by the Ethics Review Panel of the University of Luxembourg and conducted in accordance with the Declaration of Helsinki. Measures were taken to ensure that an equal number of male and female participants were recruited.

### Procedure

Participants first provided written informed consent and received standard information about the set up without being informed about the actual aim of the study (i.e., to investigate VR-induced distraction from pain). Participants then completed questionnaires (either in English or German) assessing their demographic and health status (including simulator sickness symptoms to establish a baseline), video gaming skills, emotional distress (i.e., stress, anxiety, and depression symptoms), and negative pain-related cognitions, in addition to three computerized executive function tasks.

**Figure 1:**
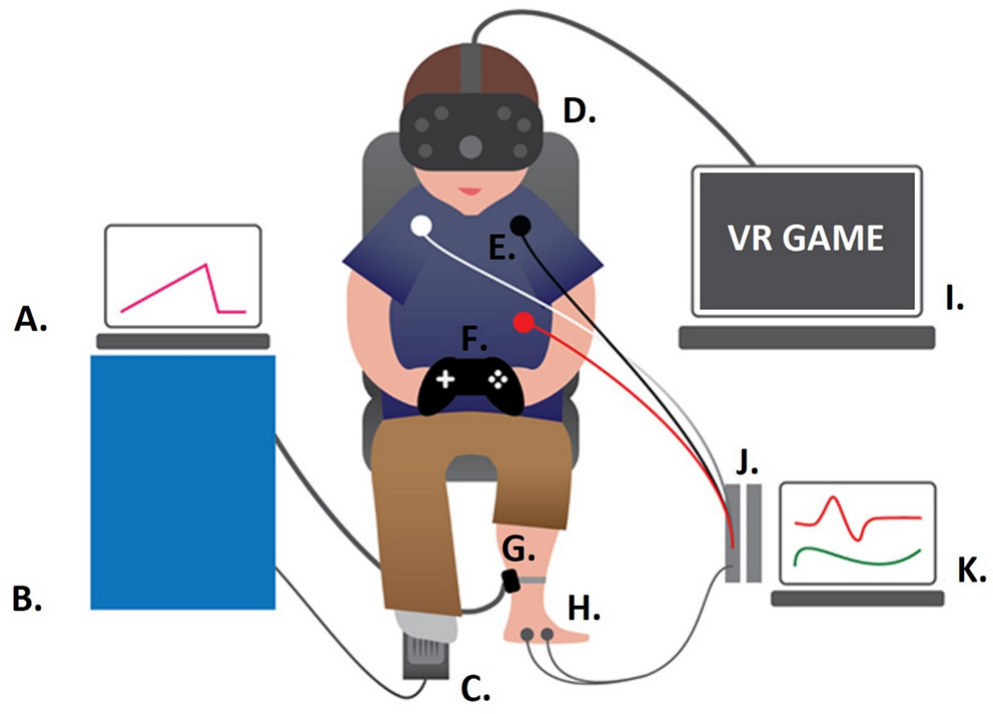
Experimental Set-Up. **(A)** Laptop operating the thermal stimulator; **(B)** PATHWAY 30 x 30 mm ATS thermal stimulator (Medoc, Ltd.); **(C)** foot switch; **(D)** *htc Vive*; **(E)** ECG electrodes; **(F)** *Xbox* controller; **(G)** thermal stimulator attached to the non-dominant leg (calf); **(H)** EDA electrodes; **(I)** gaming computer streaming *Subnautica* to the head mounted display; **(J)** *BIOPAC* modules; **(K)** computer running *AcqKnowledge* to record the psychophysiological data.

Following this, participants underwent the VR paradigm. Thermal pain thresholds were recorded during a baseline phase to familiarize them with the protocol, as well as during two interactive VR gaming conditions with different levels of cognitive load. The order of the latter two conditions was counterbalanced (see Pain Thresholds and VR Paradigm). After each of the two interactive conditions, participants completed questionnaires assessing spatial presence, simulator sickness symptoms, and perceived task difficulty. Breaks between all VR sessions lasted at least 5 min. The experimenter continuously monitored participants and, if necessary, interrupted the session if participants were showing or reporting symptoms of simulator sickness (e.g., paleness, sweating, and vertigo). We also measured participants' heart rate and electrodermal activity throughout the VR session, but only analyzed heart rate variability in the present study. The complete experimental procedure lasted approximately 100 min. For a schematic illustration of the experimental setup, see Figure 1.

### Questionnaires

#### Gaming and Virtual Reality

Gaming skill was assessed using a seven-item version of the Game Playing Skill Scale [GaPS; (25)]. Participants also indicated their average gaming time (gaming hours/day), rated their computer and VR experience (no experience, basic experience, advanced experience, expert experience) and self-evaluated their gaming expertise (no gamer, casual gamer, regular gamer, expert gamer). To assess the level of presence, i.e., the feeling of “being there” (26) in the VR, participants completed two subscales of the Spatial Presence Experience Scale (SPES) (27) after each interactive VR condition. They, furthermore, completed the Simulator Sickness Questionnaire (SSQ) (28) before the baseline condition and after each interactive VR condition. To assess the degree of perceived task difficulty, participants also rated the perceived cognitive load after each interactive condition (29).

#### Pain-Related Cognitions

The Fear of Pain Questionnaire-III [FPQ; (30)] was used to assess the participants' general fear of pain. The FPQ-III is a 30-item self-report inventory with items being rated on a 5-point scale (1 = *not at all*, to 5 = *extreme*).

The participants' level of pain catastrophizing was measured with the Pain Catastrophizing Scale [PCS; (31)]. The PCS consists of 13 items that are rated on a 5-point scale (0 = *not at all*, to 4 = *all the time*).

Attention to pain was assessed with the Pain Vigilance and Awareness Questionnaire [PVAQ; (32)], which consists of 16 items that are rated on a 6-point scale (0 = *never*, to 5 = *always*).

#### Emotional Distress

Participants were asked to rate their symptoms of emotional distress in the week prior to study participation using the Depression Anxiety Stress Scale-21 (DASS-21) (33). This scale comprises three subscales (with seven items each), providing a dimensional assessment of three components of emotional distress, including anxiety, depression, and stress. Responses were given on a 4-point scale (from 0 = *not at all/never*, to 3 = *most of the time/almost always*).

### Executive Functioning Tasks

Participants completed three executive functioning tasks that were implemented in the Psychology Experiment Building Language (*PEBL)* (34).

#### Corsi Block Tapping Task

The Corsi block tapping task was used to assess visuo-spatial working memory (WM) (35). In the computerized version used in the present study, participants were presented with an array of nine blocks (squares) and in each trial a sequence of squares was highlighted, one after the other. Participants were required to memorize and reproduce the sequence in the same (forward condition) or reversed order (backward condition), by clicking on the squares. The initial sequence included only two squares and was increased by one square every third trial up to a maximum of nine squares. The task ended once two trials of the same sequence length were recalled incorrectly. The main outcome variable for both conditions was the block span, i.e., the longest length at which at least one sequence was correctly recalled.

#### Flanker Task

A modified version of the Eriksen flanker task by Stins et al. (36) was used to assess interference control and selective attention, i.e., the ability to filter out competing information by concentrating on one stimulus and ignoring the surrounding stimuli (37). In the present task, five horizontally aligned arrows appeared in the middle of a screen. Participants were asked to identify the direction of the target (centre) arrow, which either pointed in the same direction as the flanking arrows (congruent condition) or in the opposite direction (incongruent condition). In total, participants completed 80 trials (40 congruent, 40 incongruent), following a practice run of eight trials. To calculate the flanker effect, the mean reaction time (RT) of congruent trials was subtracted from the mean RT of incongruent trials. Higher scores in the flanker task indicate less efficient interference control and worse selective attention.

#### Go/NoGo Task

The go/nogo task (38) assesses prepotent response inhibition abilities, i.e., the ability to suppress inappropriate or irrelevant actions (39). The present task was designed after Bezdjian et al. (40) and required participants to respond to the letter “P” with a button press and to withhold their response to the letter “R.” Participants completed two blocks (in the second block the *go* and *nogo* stimuli were reversed) containing 160 trials each, following a practice run of 10 trials. Response inhibition was quantified by computing the percentage of commission errors, i.e., presses in response to *nogo* stimuli. A lower percentage indicates a better response inhibition capacity.

### Pain Thresholds

Pain thresholds were measured using a PATHWAY 30 x 30mm ATS thermal stimulator “thermode” (Medoc Ltd.), which was attached to the lower calf of the participant's non-dominant leg. The baseline temperature of the thermode was set to 32°C and increased at a slope of 0.5°C/s to a maximum of 50°C. As soon as the participants perceived the temperature as painful, they were instructed to press a foot switch (placed under the dominant leg), which triggered a rapid return to baseline temperature at a slope of 10°C/s. The temperature at the time of the participant's response was recorded as the pain threshold. The inter-stimulus interval varied between 45 and 50 seconds. In total, pain thresholds were measured 35 times (five times during a baseline phase), 15 times in the low cognitive load condition (LLC), and 15 times in the high cognitive load condition (HLC).

### Electrocardiography Data

Heart rate was assessed continuously using a three-lead electrocardiogram (ECG; electrodes were attached to the chest following Einthoven's triangle), recorded at a 1 kHz sampling rate using the MP150 system and AcqKnowledge software 4.4.1 (Biopac Systems Inc.).

### VR Paradigm

Participants were equipped with a head-mounted display (HMD; *htc* VIVE), headphones and an Xbox controller. In the interactive VR conditions, participants played the video game *Subnautica* (Unknown Worlds Entertainment, Inc.), an underwater exploration game played from a first-person perspective of a scuba diver (note that participants could only see the scuba diving gloves of their avatar). For the present study, several gaming settings were changed enabling unlimited oxygen supply, invincibility, and reduced swimming speed.

Pain thresholds were assessed during the baseline and two interactive conditions with either a low cognitive load (LLC) or a high cognitive load (HLC). During the baseline, participants sat stationary and passively in the virtual environment on an inflatable island surrounded by the sea and sky. This condition served as an opportunity for participants to familiarize themselves with the VR and the pain threshold procedure. The LLC consisted of a simple navigation task: participants had to follow a pipeline leading through the underwater environment. In the HLC, participants had to follow the same underwater route, but in addition, they were asked to memorize a sequence of eight single digit numbers that were displayed at fixed intervals along the pipeline. Note that the HLC differed from the LLC only in terms of working memory demands (and beacons used to present the digits), allowing a direct comparison of the effects of additional cognitive load on VR-related hypoalgesia. Recall of the digit sequence was assessed directly after completing the session and quantified using the Damerau-Levensthein distance (41) that indicates how many operations are needed to transform one sequence into another (e.g., the reported digit sequence into the correct one). Higher values indicate more operations, i.e., a greater distance, and thus lower memory performance.

### Data Preparation

#### Pain Thresholds

We excluded pain thresholds of 37°C and lower [most likely representing an accidental foot press (42)] and of 50°C (i.e., trials without a registered response). This resulted in removing a total of 23 thresholds from 13 participants. We also excluded one participant from all analyses who had an average pain threshold of *M* = 49.71°C in the baseline condition, and of *M* = 49.99°C in the LLC and HLC, exceeding 3 SD.

#### Heart Rate Variability

ECG data were processed and analyzed with the PhysioData Toolbox [Version 0.5.0; (43)]. Two participants had to be excluded completely from the analyses due to anomalies (e.g., premature heart beats). Technical complications (e.g., detached electrodes) led to the rejection of two measurements for the LLC and one for the HLC. We extracted four metrics to assess heart rate variability in the time and frequency domain for the duration of each condition (LLC_duration_: *M* ±*SD* = 677.79 ± 12.49 s, HLC_duration_: *M* ±*SD* = 696.61 ± 48.20 s). We computed the standard deviation of the inter beat intervals of normal sinus beats (detrended SDNN) and the root mean square of successive differences between normal heartbeats (detrended RMSSD) in milliseconds as time domain indices of vagally mediated changes in HRV (44). Low frequency (LF; Hz range: 0.04–0.15 Hz) and high frequency (HF; Hz range: 0.15–0.4 Hz) power (in ms^2^) were used as frequency domain measures. The Lomb-Scargle method was used to estimate the Power Spectral Density of the interbeat interval time series to compute LF and HF power (43).

### Statistical Analyses

Statistical analyses were performed using SPSS 26 (IBM SPSS Statistics). The difference in pain thresholds between the HLC and LLC (i.e., *PainLoad*) were assessed with a repeated measures ANOVA, and the predictive power of emotional distress, pain-related cognitions and cognitive skills with a regression analysis on (the difference in) pain thresholds. The association between changes in HRV and pain thresholds were assessed using two-tailed Spearman correlations with bias-corrected and accelerated bootstrapping (1,000 samples) as a Spearman correlation does not require that the data is normally distributed and is relatively robust against outliers (45).

Power analysis with G^*^Power 3.1 (46, 47) for a repeated measures ANOVA with two measurements and an assumed medium effect size of *f* = 0.15, and an α = 0.05, showed that a total sample size of 90 participants is sufficient to obtain a power of 0.80. For a multiple linear regression with five tested predictors, and an assumed effect size of *f*^2^ = 0.15, α = 0.05 and a power of 0.80, a sample size of 92 participants is necessary. A sample size of 67 or 84 participants is sufficient to obtain a power of 0.80 for a one-tailed or two-tailed correlation, respectively, assuming a correlation effect size of ρ = 0.30 for H_1_ and ρ = 0.0 for H_0_.

## Results

### Descriptive Statistics

A total of 101 participants were recruited and tested for the study. The data of 11 participants were excluded from the analyses due to discontinuation of the study due to simulator sickness (*n* = 4), an average pain threshold close to the limit of 50°C (*n* = 1; see Data Preparation), technical problems with the thermal stimulator (*n* = 1), and self-reported acute or chronic pain (*n* = 5). Thus, the final sample consisted of 90 participants (50% identified as male; 74.4% German speaking; 84.4% students) with a mean age of 23.46 years (*SD* = 3.28).

More than half of the participants reported to be advanced users of computer equipment (65.56 %), but only 8.89 % stated to have advanced experience using VR and 56.67 % had no prior VR experience at all. Average daily time spent on video games was <1 h (*M* = 0.96 hours, *SD* = 1.47), and 43.33 % stated to be current non-players. About half of all participants (46.7%) completed the HLC first, the other half started with the LLC. Descriptive statistics of the psychological and cognitive (performance) measures are given in Table 1. Task-related and HRV measures for the LLC and HLC are reported in Table 2. Tests of normality for all variables of interest can be found in the Supplementary Materials (see Supplementary Table S1).

**Table 1:** Psychometric characteristics of the sample.

**Variables**	**Mean**	**Standard deviation**	**Sample scale range**
**Executive functions**			
Corsi forward (block span)	6.48	1.36	2–9
Corsi backward (block span)	6.69	1.50	1–9
Flanker effect (ms)	41.63	20.22	8.35–127.95
Go/NoGo (percentage)^a^	31.36	15.44	6.25–75.00
**Pain-related cognitions**			
FPQ-III	82.33	17.63	42–127
PCS	17.96	9.43	0–37
PVAQ	35.77	12.75	11–68
**DASS-21**	11.17	8.96	0–46
Depression	3.79	3.84	0–16
Anxiety	2.51	2.99	0–14
Stress	4.87	3.78	0–19
**Memory task performance (HLC)^b^**	1.93	2.12	0–8

*FPQ-III, Fear of Pain Questionnaire-III; PCS, Pain Catastrophizing Scale; PVAQ, Pain Vigilance and Awareness Questionnaire; DASS-21, Depression Anxiety Stress Scale; HLC, high load condition.*

a *Percentage of commission errors; based on 89 as the data for one participant could not be retrieved.*

b *Based on 86 as four participants did not complete the memory task before the end of the pain threshold procedure*.

**Table 2:** Task-related and HRV measures.

**Variable**	**Mean ± standard deviation** **LLC**	**Mean ± standard deviation** **HLC**	**Sample scale range**	**t-statistic**	***p***
**Task-related measures**					
Perceived task difficulty	1.78 ± 0.99	4.64 ± 1.57	1–8	−15.60	<0.001
Spatial presence	3.56 ±0.79	3.35 ±0.84	1–5	2.94	0.004
Simulator sickness	1057.54 ± 277.77	1059.85 ± 274.68	812.63–2254.85	−0.11	0.909
**HRV measures^a^**					
RMSSD (detrended)	38.19 ± 20.72	34.95 ± 17.22	-	3.29	0.001
SDNN (detrended)	41.78 ± 15.84	38.97 ± 13.12		3.78	<0.001
LF power	1108.60 ± 812.50	974.31 ± 664.40	-	2.89	0.005
HF power	718.62 ± 901.48	544.30 ± 545.32	-	3.19	0.002

a *Based on 85 participants as five participants were excluded from the analysis of the HRV metrics due to technical problems and premature heart beats. RMSSD, root mean square of the successive differences; SDNN, standard deviation of normal-to-normal R-R intervals; LF, low frequency; HF, high frequency*.

### Effect of Cognitive Load on Pain Thresholds

We first explored whether *age* and gaming skills (*GaPS scores*) were correlated with the difference in temperature (pain thresholds) between the HLC and LLC (i.e., *PainLoad*) using two-tailed Spearman correlations with bootstrapping and tested whether *PainLoad* was significantly different for *gender* and *task order* using independent samples *t*-tests. All correlations were non-significant (*p* > 0.324). We also found no significant differences for *gender* (*p* = 0.392) but a marginally significant effect for *task order* (*p* = 0.068).

A repeated measures ANOVA with the within-subject factor *cognitive load* (LLC vs. HLC), showed no main effect of *cognitive load, F* (1, 89) = 1.23, *p* =0.271, $n_{p}^{2}$ =0.014. (Note that the main effect of *cognitive load* became significant, *F* (1, 88) = 4.44, *p* =0.038, $n_{p}^{2}$ =0.048, when adding *task order* as covariate. However, the overall difference in °C between the HLC and LLC was small, with a mean difference of M = 0.12, SE = 0.10°C). Closer inspection revealed that only 51.1% of the participants experienced a positive effect of cognitive load, i.e., higher pain thresholds in the HLC compared to the LLC (mean difference: *M* = 0.66, *SD* = 1.07°C) whereas the remaining 48.9% participants showed the opposite effect of higher pain thresholds in the LLC than in the HLC (mean difference: *M* = −0.45, *SD* = 0.39°C).

A chi-square goodness of fit test revealed no significant difference in *gender* distribution between the participants who benefited more from the LLC (47.83% identified as male) or HLC (52.27% identified as male), *p* = 0.673. In line with the results reported above, we found a marginally significant effect of *task order, X*^2^ (1, 90) = 3.67, *p* = 0.055, indicating that participants who had completed the LLC first, tended to benefit more often from the LLC than the HLC (63.64%) and participants who had completed the HLC first, tended to benefit more often from the HLC than the LLC (56.52%). We found no significant differences in *age* (*p* = 0.901) or *GaPS scores* (*p* = 0.590) between groups, as tested with independent samples *t*-tests.

**Figure 2:**
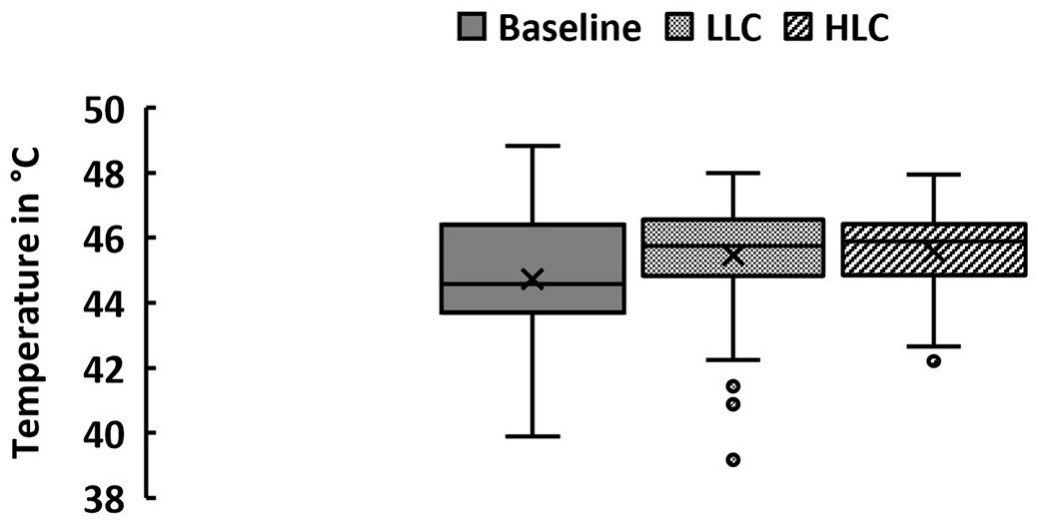
Pain thresholds. Pain thresholds were assessed during a baseline condition (in a static VR environment) and two interactive conditions with a low cognitive load (LLC) and a high cognitive load (HLC) while participants (male = 45, female = 45) were immersed in VR. *M* ±*SD*: Baseline: 44.73 ± 1.81°C, LLC: 45.47 ± 1.53°C, HLC: 45.58 ± 1.20°C. Note that the “x” in the middle of the boxplot denotes the mean whereas the horizontal line denotes the median. The whiskers denote the minimum and maximum values. Outliers (i.e., data points that are 1.5 times larger or smaller than the interquartile range) are represented by dots.

**Figure 3:**
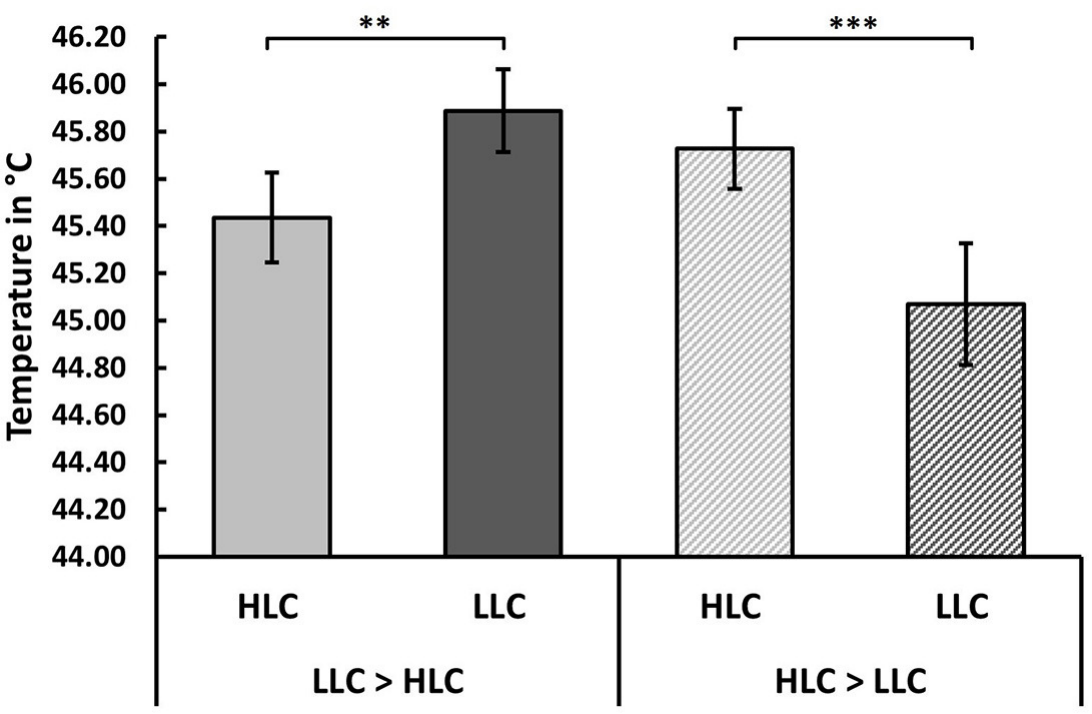
Effect of cognitive load on pain thresholds. Participants could be subdivided into two subgroups, showing distinct responses to the high load task (HLC). About half of all participants (51.1%) showed an increased pain threshold in the HLC relative to the LLC, whereas the remaining 48.9% exhibited a higher pain threshold in the LLC relative to the HLC. Bar charts illustrate the average pain thresholds for both conditions for each subgroup. Error bars represent the standard error of the mean (SEM). **p < 0.01, ***p < 0.001.

To assess whether cognitive load significantly modulated pain thresholds in these two subgroups, we ran another repeated measures ANOVA with *cognitive load* as within-subject factor, and *subgroup* (HLC>LLC or LLC>HLC) as between-subject factors. This ANOVA revealed no significant main effect of *cognitive load* [*F* (1, 88) = 1.42, *p* = 0.236, $n_{p}^{2}$ = 0.016]. However, we observed a significant interaction between *cognitive load* and *subgroup* [*F* (1, 88) = 41.85, *p* < 0.001, $n_{p}^{2}$ =0.322]. (Note that adding *task order* as a covariate to the ANOVA did not change the significance of this interaction, *F* (1, 87) = 37.76, *p* < 0.001, $n_{p}^{2}$ = 0.303). Bonferroni-corrected *post hoc* tests showed that participants in the HLC>LLC group showed a slightly larger increase in pain thresholds from the LLC to the HLC [*F* (1, 88) = 30.02, *p* < 0.001, $n_{p}^{2}$ = 0.254] than the other subgroup from the HLC to the LLC [*F* (1, 88) = 13.62, *p* < 0.001, $n_{p}^{2}$ = 0.134]. Boxplots for each condition can be found in Figure 2, and bar charts of the average pain thresholds in each condition and for each subgroup in Figure 3.

### Predictors of the Effect of Cognitive Load

To estimate the likelihood of participants to benefit more from the HLC than the LLC (or vice versa) based on self-reported emotional distress symptoms, pain-related cognitions, and executive functions, we conducted a hierarchical binary logistic regression (computing bias-corrected and accelerated 95% confidence intervals using bootstrapping (1,000 samples), with *subgroup* (HLC> LLC or LLC>HLC) as dependent variable. (Note that we decided to conduct a logistic regression instead of a multiple linear regression as this facilitates the interpretation of our finding that participants responded differently to the increased cognitive load. A *post hoc* analysis of the achieved power for the logistic regression is reported below).

To control for potential effects of *task order*, we entered this variable at stage one of the model. At stage two, we entered the total DASS-21 score and PCS score as measures of emotional distress and pain-related cognitions, and the performance measures of the executive functioning tasks (block span in the Corsi forward condition, flanker effect, percentage of commission errors in the go/nogo task). We did not control for gaming skills here, as adding GaPS scores as a covariate to the ANOVA showed no influence of gaming skills on pain thresholds.

We chose to enter PCS as a pain-related cognition to limit the number of predictors and to reduce multicollinearity. The total PCS score in the present study was highly correlated with the total FPQ-III score (*r*_*s*_ = 0.627) and PVAQ score (*r*_*s*_ = 0.621) and has been shown in several previous studies to affect the magnitude of distraction from pain (18, 48, 49). All three measures of the executive functioning tasks were uncorrelated with one another (all *p*s > 0.055).

Screening the five tested predictors for multivariate outliers by computing Mahalanobis distance revealed that one participant was a multivariate outlier, and hence removed from the regression model. We removed one additional participant from the regression model with a Cook's distance of 0.714 (compared to an average distance of 0.084 ± 0.088 SD), who represented an unduly influential data point. As the percentage of commission errors in the go/nogo task could not be retrieved for one participant, the overall regression model was thus based on 87 participants.

Results revealed that emotional distress and the block span in the forward condition of the Corsi block tapping task were significant predictors of the likelihood to benefit more, or less, from the HLC relative to the LLC with an overall classification accuracy of 67.80%, after controlling for the effects of *task order*. For a one unit increase in the DASS-21 score, the odds of benefiting more from the HLC than the LLC decreased (OR: 0.916). A one unit increase in the forward condition of the Corsi block tapping task, on the other hand, was associated with an increased odds ratio to benefit more from the HLC than the LLC (OR: 1.566); however, bias-corrected and accelerated bootstrap intervals (BCa 95% CI) suggest that performance in the Corsi task is not a robust predictor. See Table 3 for a comparison of both models and Table 4 for the classification accuracy of the model.

**Table 3:** Hierarchical binary logistic regression model.

**Variable**	**Model 1**				**Model 2**			
	***B***	**Odds ratio**	***p***	**BCa 95% CI**	***B***	**Odds ratio**	***p***	**BCa 95% CI**
Constant	−0.351	0.704	0.246	[−1.003 0.245]	−4.685	0.009	0.001	[−8.403 –2.576]
Task order^a^	0.798	2.220	0.073	[−0.095 1.726]	1.496	4.465	0.004	[0.063 3.983]
DASS-21					−0.087	0.916	0.008	[−0.165 −0.040]
PCS					0.022	1.022	0.514	[−0.053 0.127]
Corsi forward (block span)					0.449	1.566	0.011	[−0.027 1.073]
Flanker effect					0.023	1.023	0.126	[−0.018 0.067]
Go/NoGo (commission errors)					0.023	1.023	0.206	[−0.022 0.106]
Nagelkerke pseudo *r*^2^	5.1%				27.5%			
χ^2^	3.379, df = 1, *p* = 0.066				20.094, df = 6, *p* < 0.003			

*The regression model is based on 87 participants (HLC>LLC: 44; LLC > HLC: 43). Internal coding: LLC (low load condition) > HLC (high load condition) is group: 0, HLC > LLC is group: 1.*

a *Task order: 1 = HLC, then LLC; 2 = LLC, then HLC. DASS-21, Depression Anxiety Stress Scale; PCS, Pain Catastrophizing Scale*.

**Table 4:** Classification table.

		**Predicted**		**Correct %**
		**LLC > HLC**	**HLC > LLC**	
Observed	LLC > HLC	28	15	65.1
	HLC > LLC	13	31	70.5

*LLC, low load condition; HLC, high load condition*.

*Post hoc* power analysis for the logistic regression in G^*^Power 3.1 indicated an achieved power of 0.84 for the effect of the DASS-21 score (OR for standardized DASS-21: 0.463; assumed Pr_H0_(Y=1|X=1) = 0.5; *R*^2^ = 0.14; α = 0.05), and a power of 0.72 for the effect of block span in the Corsi forward condition (OR for standardized block span: 1.843; assumed Pr_H0_(Y=1|X=1) = 0.5; *R*^2^ = 0.04; α = 0.05).

Given that the block span in the Corsi forward condition was only weakly correlated with the block span in the backward condition (*r*_*s*_ = 0.275), we conducted another exploratory logistic regression replacing the block span in the forward condition with the block span in the backward condition. However, the regression model showed that the block span in the Corsi backward condition (i.e., visuo-spatial working memory) was not predictive of the group affiliation [*B* = 0.088, *SE* = 0.063, *p* = 0.176, 95%CI(−0.024 0.236)].

### Heart Rate Variability and Cognitive Load

In a first step, we computed the differences in HRV measures (RMSSD, SDNN, LF, and HF power) between the HLC and LLC (HLC-LLC) and established that the difference scores were not significantly different for *gender* or *task order* using independent sample *t*-tests (all *p*s > 0.061). Subsequently, we ran two-tailed Spearman rank correlations with bootstrapping (1,000 samples), computing bias-corrected and accelerated 95% confidence intervals (BCa 95% CI) between the difference in HRV measures and *PainLoad* to assess whether a relatively higher HRV in the HLC relative to the LLC would reflect higher pain thresholds in the HLC compared to the LLC. Results revealed only marginally significant (*p* < 0.100) positive correlations between the differences in RMSSD, SDNN and *PainLoad*, i.e., higher pain thresholds in the HLC compared to the LLC were associated with a higher RMSSD [*r*_*s*_ = 0.189, *p* = 0.083, 95%CI(−0.025 0.377)] and SDNN [*r*_*s*_ = 0.187, *p* = 0.086, 95%CI(−0.018 0.393)] in the HLC relative to the LLC.

### Heart Rate Variability and Emotional Distress

Given that HRV signals relaxation and sympathetic/parasympathetic balance (21), we explored whether participants who reported to be more distressed, showed a greater degree of relaxation in the LLC than in the HLC, possibly explaining why they benefited more from the LLC than from the HLC. For this purpose, we conducted one-tailed Spearman correlations (using bootstrapping with 1,000 samples, BCa 95% CI). This revealed a significant negative association between the total DASS-21 score and the difference in RMSSD between the HLC and LLC (note however that the 95%CI indicates no robust association). Correlations with the DASS subscales showed that self-reported anxiety symptoms were particularly strongly related to the difference in HRV measures, i.e., participants reporting more anxiety (and stress) tended to show lower RMSSD, SDNN and LF power in the HLC compared to the LLC (see Table 5).

**Table 5:** One-tailed spearman correlations between distress symptoms and differences in HRV between the HLC and LLC.

**Variable**	**Difference in RMSSD (detrended)**		**Difference in SDNN (detrended)**		**Difference in LF power**		**Difference in HF power**	
	***r_s_***	**95% BCa CI**	***r_s_***	**95% BCa CI**	***r_s_***	**95% BCa CI**	***r_s_***	**95% BCa CI**
DASS-21 (total)	−0.211^*^	[−0.415 0.002]	−0.176	[−0.347 0.001]	−0.145	[−0.335 0.054]	−0.090	[−0.294 0.123]
Depression	−0.071	[−0.304 0.138]	−0.077	[−0.269 0.125]	−0.064	[−0.285 0.152]	0.003	[−0.22 0.232]
Anxiety	−0.238^*^	[−0.430– 0.027]	−0.213^*^	[−0.408 −0.011]	−0.215^*^	[−0.428 −0.002]	−0.133	[−0.347 0.090]
Stress	−0.193^*^	[−0.381 0.001]	−0.163	[−0.353 0.030]	−0.125	[−0.313 0.072]	−0.050	[−0.256 0.153]

*Based on 85 participants as five participants were excluded from the analysis of the HRV metrics due to technical problems and premature heart beats. LLC, low load condition; HLC, high load condition; DASS-21, Depression Anxiety Stress Scale; RMSSD, root mean square of the successive differences; SDNN, standard deviation of normal-to-normal R-R intervals; LF, low frequency; HF, high frequency.*

* *p < 0.05*.

## Discussion

In the present study, we investigated the effect of an additional cognitive load in a virtual reality game on modulating pain thresholds and the role of individual differences. Prima facie, we found no effect of cognitive load on pain thresholds when assessing the whole sample, even though significant differences in task difficulty ratings, spatial presence and HRV measures suggest that the two conditions were sufficiently distinct to result in differences on the cognitive and psychophysiological level. Closer examination revealed that the lack of a modulatory effect resulted from two different response types to the additional cognitive load in our sample. While about half of all participants showed higher pain thresholds in the more challenging high load condition (sensory immersion plus cognitive memory challenge), the other half benefitted more from the low load condition (sensory immersion only). These two subgroups emerged independently of participant gender or gaming skills but differed significantly in self-reported emotional distress (depression, anxiety, and stress symptoms) and visuospatial short-term memory (block span in the forward condition of the Corsi block tapping task).

Participants reporting more distress had significantly lower odds to benefit from the additional cognitive challenge (HLC), possibly because the memory task in the HLC exceeded the cognitive resources that more distressed participants were willing to invest in completing the memory task (50). More distressed participants may have also perceived the low load condition as more entertaining than the high load condition, a factor that has been shown to modulate the efficacy of VR-related distraction from pain in previous studies (51, 52), but that we did not assess in the present study. This explanation is also in line with research showing that it is the individual's absorption in the distracting task rather than the type of task that is determining the efficacy of distraction (53). Another potential moderator for the differential influence of increased cognitive load on VR-related hypoalgesia could be individual differences in situational anxiety. It seems, e.g., plausible that exploring the underwater environment during the LLC may have led to stress and anxiety relieving effects (54, 55). These effects may have been more pronounced for participants who had higher levels of distress whereas participants low in emotional distress may have not been able to further down-regulate their distress levels. This explanation is partly supported by previous research showing that VR-induced increases in heat pain tolerance are likely driven by the degree of relaxation (21). In agreement with this, exploratory analyses showed that participants who reported more emotional distress, in particular higher levels of anxiety, tended to show higher RMSSD in the LLC than in the HLC in the present study. However, the overall strength of the associations was weak and future studies should directly assess the degree of relaxation and situational anxiety by self-report measures administered before and after the intervention.

This finding could have important implications for clinical settings; patients who seek medical attention for (pain) symptoms or who undergo medical treatment, such as surgeries, are likely to experience high levels of distress (56, 57) and thus, it seems plausible that effect sizes may be even more pronounced in patients. Importantly, a comparison of our data to intensity and unpleasantness ratings of heat stimuli within the same temperature range from other studies in our lab (see Supplementary Table S2) suggests that an increase of 0.5°C in intensity was associated with a 5–13% increase in subjective pain ratings. This indicates that the changes in heat pain thresholds observed in the present study are likely to have clinical relevance.

We also found that participants with a better visuospatial short-term memory were more likely to benefit from the additional cognitive load (although 95% BCa confidence intervals indicate that this association is not very robust, probably partly due to little heterogeneity in cognitive performance in our sample of healthy young adults). Given that the HLC comprised a visual (digit-based) memory task, the observed association is not surprising and emphasizes the need to assess the cognitive components of VR environments/tasks when using VR as a pain intervention tool and to carefully match them to the participant's cognitive abilities, especially in clinical settings where executive functions may be (temporarily) hampered, e.g., due to side effects from medications (58).

Importantly, we quantified the effect of an additional cognitive load not by relying on a single retrospective self-report (59–61), but by assessing heat pain thresholds in regular intervals while participants were immersed in VR. This makes the data less prone to be biased by social desirability or the elapsed time between the nociceptive stimulation and its evaluation (62, 63). We also chose tasks that are unlikely to be confounded by previous gaming experience or motor coordination skills (as all tasks were self-paced and required minimal and easy use of the controller).

Taken together, our results clearly suggest that the analgesic effects of VR cannot be fully explained by distraction, i.e., competition for limited shared attentional resources (5), as such an explanation should have entailed a near-systematic reduction in pain thresholds in the high load, relative to the low load, condition. Rather, our study adds to a complex picture, suggesting that VR-related analgesia may involve various underlying mechanisms, such as affect modulation in addition to attentional diversion (4). Moreover, our findings also indicate that individual differences in cognitive and affective state shape how individuals respond to these mechanisms.

Despite these strengths, our study has a few limitations. First, our participants were healthy pain-free young adults (mostly students) with good executive functions, limiting the generalizability of the results to other populations. Although visual comparisons show that pain thresholds in the interactive VR conditions were significantly increased when compared to the passive baseline condition (see Figure 2), our study design does not allow to draw any concrete conclusions about the efficacy of VR in distracting from pain in general. A more complex design as suggested by Colloca et al. (21) would allow to further disentangle the effects of cognitive load on pain thresholds, albeit this would have likely gone beyond the scope of the present study. Nevertheless, we encourage future studies to replicate our findings including a non-VR baseline condition.

In addition, the specific characteristics of our study design may have hampered our ability to assess the role of cognitive inhibition and selective attention abilities in distraction from pain. In particular, the high load condition required participants to shift their attention from one task (e.g., navigation) to the other (e.g., memorizing the digit span) and thus, did not allow to selectively attend to one task. Switching between tasks has also decreased perceived spatial presence (as evidenced by significantly lower self-reported spatial presence in the HLC relative to the LLC). Future studies that investigate the effects of cognitive load in VR should consider using more gamified approaches of implementing the cognitive challenge in the VR environment to avoid potential breaks in presence. However, researchers should at the same time ensure comparable conditions with regard to the required coordination and motor skills. In addition, it may be helpful to explicitly assess the participants' level of fun and perceived mental workload in future studies [e.g., with the NASA Task Load Index; (64)] to explain individual differences in the efficacy of VR to distract from pain.

Overall, our findings suggest that a VR game with an additional cognitive load does not automatically result in reduced pain sensitivity. Rather than using a “one size fits all” approach, our study emphasizes the need to consider individual differences, especially affective factors, when choosing VR as an intervention tool for pain treatment to maximize its therapeutic effects. Assessing the role of individual differences may also prove helpful in accounting for differences in study findings that cannot be explained by VR configuration factors alone.

## Data Availability Statement

The original contributions presented in the study are included in the article/Supplementary Materials, further inquiries can be directed to the corresponding author.

## Ethics Statement

The studies involving human participants were reviewed and approved by Ethics Review Panel at the University of Luxembourg. The patients/participants provided their written informed consent to participate in this study.

## Author Contributions

The study was designed by EH, LB, and KMR and implemented by KB, EH, LB, and KMR, who were also involved in the data collection (KB contributed the majority to the data collection). KB wrote the first draft of the manuscript that was subsequently revised by the other authors. All authors contributed to the data analyses, discussion of the results, manuscript revision, read, and approved the submitted version.

## Funding

The study was funded by the Doctoral School of Humanities and Social Sciences at the University of Luxembourg with a research grant awarded to EH, LB, and KMR.

## Conflict of Interest

The authors declare that the research was conducted in the absence of any commercial or financial relationships that could be construed as a potential conflict of interest.

## Publisher's Note

All claims expressed in this article are solely those of the authors and do not necessarily represent those of their affiliated organizations, or those of the publisher, the editors and the reviewers. Any product that may be evaluated in this article, or claim that may be made by its manufacturer, is not guaranteed or endorsed by the publisher.
