# Supplementary material for: When Less Is More: Investigating Factors Influencing the Distraction Effect of Virtual Reality From Pain
Source: Front Pain Res (Lausanne). 2022 Jan 5;2:800258. doi: 10.3389/fpain.2021.800258 (PMC8915653; doi:10.3389/fpain.2021.800258)
Supplement: Supplementary file 1 [file Table_1.DOCX]

Supplementary Material

**Supplementary Table 1.** Tests of normality for the variables of interest.

|  | Kolmogorov-Smirnov | | |
| --- | --- | --- | --- |
| Variables | Statistic | df | Significance |
| Age | 0.155 | 90 | 0.000 |
| Gaming skills (GaPS) | 0.140 | 90 | 0.000 |
| DASS-21 (total) | 0.184 | 90 | 0.000 |
| DASS-21 (depression) | 0.237 | 90 | 0.000 |
| DASS-21 (anxiety) | 0.257 | 90 | 0.000 |
| DASS-21 (stress) | 0.202 | 90 | 0.000 |
| PCS (total) | 0.095 | 90 | 0.045 |
| FPQ-III (total) | 0.089 | 90 | 0.079 |
| PVAQ (total) | 0.078 | 90 | 0.200 |
| Corsi forward (block span) | 0.257 | 90 | 0.000 |
| Corsi backward (block span) | 0.254 | 90 | 0.000 |
| Flanker effect (ms) | 0.081 | 90 | 0.193 |
| PainLoad (HLC-LLC) | 0.198 | 90 | 0.000 |
| RMSSD (detrended) | 0.139 | 85 | 0.000 |
| SDNN (detrended) | 0.127 | 85 | 0.002 |
| LF power | 0.195 | 85 | 0.000 |
| HF power | 0.260 | 85 | 0.000 |

*Note.* DASS-21 = Depression Anxiety Stress Scale; PCS = Pain Catastrophizing Scale; FPQ-III = Fear of Pain Questionnaire-III; PVAQ = Pain Vigilance and Awareness Questionnaire; PainLoad = difference in heat pain thresholds between the high load (HLC) and low load (LLC) condition; RMSSD = root mean square of the successive differences; SDNN = standard deviation of normal-to-normal R-R intervals; LF = low frequency; HF = high frequency.
