# Supplementary material for: When Less Is More: Investigating Factors Influencing the Distraction Effect of Virtual Reality From Pain
Source: Front Pain Res (Lausanne). 2022 Jan 5;2:800258. doi: 10.3389/fpain.2021.800258 (PMC8915653; doi:10.3389/fpain.2021.800258)
Supplement: Supplementary file 2 [file Table_2.DOCX]

Supplementary Material

**Supplementary Table 2.** Calibration data for 16s heat stimuli.

| Temperature | Intensity scale | |  | Unpleasantness scale | |
| --- | --- | --- | --- | --- | --- |
| (in °C) | Ratings | Change in % |  | Ratings | Change in % |
| 45 | 68.98 | 7.75 |  | 83.40 | 5.645 |
| 45.5 | 84.47 | 7.59 |  | 94.69 | 6.135 |
| 46 | 99.65 | 13.79 |  | 106.96 | 12.460 |
| 46.5 | 127.22 |  |  | 131.88 |  |

*Note.* Data of n = 32 healthy young participants (18-35 years old) who rated the intensity and unpleasantness of a series of heat stimuli on their left forearm that lasted for 16s (with a plateau phase of 10s) on 200-point intensity and unpleasantness scales (0: not warmth, 100: just painful; 200: extremely painful).
